# The insurance of climate-related physical risks: challenges and opportunities for actuaries

**DOI:** 10.1007/s13385-026-00462-x

**Published:** 2026-09-30

**Authors:** Hansjörg Albrecher, Stéphane Loisel

**Affiliations:** 1 Department of Actuarial Science, Faculty of Business and Economics and Swiss Finance Institute and Expertise Center for Climate Extremes (ECCE), University of Lausanne, UNIL-Dorigny, CH-1015 Lausanne, Switzerland; 2 Lirsa, Efab, Conservatoire National des Arts et Metiers, 292 rue St Martin, F-75003 Paris, France

**Keywords:** Insurance, Natural catastrophes, Climate change, Physical risk, Prevention, Insurance regulation

## Abstract

We examine the key challenges associated with insuring climate-related risks in the context of a changing climate. Adopting a balance-sheet perspective on insurance company management, we analyze the concept of insurability and how it may be reshaped by climate change. Focussing on non-life perils, we further explore the roles of regulation, risk prevention and mitigation in addressing these issues, and discuss a range of potential solutions – and limitations – that emerge. These challenges also open up interesting opportunities for both academic research and practical innovation within the insurance industry.

## Introduction

In the last few years, there has been striking evidence that global warming is changing the atmospheric conditions significantly, leading to an increased frequency of extreme weather events, see e.g. the IPCC Sixth Assessment Report (AR6) published by the Intergovernmental Panel on Climate Change of the United Nations and the World Meteorological Organization, Seneviratne et al. [150]. The consequences of climate change are manifold for different parts of society, and also the insurance industry faces considerable challenges in this respect. As noted for example by the Financial Stability Board [68], on the one hand, insurers are exposed to *transition risk*, which refers to the financial and strategic risks that come along with moving toward a low-carbon, sustainable economy. On the other hand, they face *physical risks* stemming from the direct impact of climate change, including the increasing frequency and severity of natural catastrophes (NatCat) such as floods, storms, and heatwaves. For (re)insurance companies that underwrite NatCat-related risks, climate change directly affects the structure of their insurance liabilities. Accordingly, adjusting premiums and required solvency capital levels is essential to ensure the sound operation of their business. The year 2024 was the fifth consecutive year in which the global insured NatCat losses exceeded 100 billion USD, and 2025 will also exceed that threshold (with economic losses estimated about three times that amount, leaving a considerable insurance gap), see e.g. [11, 110, 155, 166]. In addition to changes in hazard, NatCat insurance losses have risen substantially due to increasing exposure associated with economic and population growth, asset concentration in risk-prone regions, and expanding insurance coverage, such that the extent to which observed loss trends can be robustly attributed to anthropogenic climate change remains actively debated (see, e.g., [79, 129, 130]). Recent industry and research assessments indicate that a substantial portion of the observed increase in global insured NatCat losses is attributable to changing climate conditions ( [141], e.g., estimates about one third of the increase of natural hazard insured losses over the period 2002–2022 being linked directly to anthropogenic climate change). Long-running assessments of NatCat losses of leading reinsurers in this domain (see, e.g., [110, 155]) similarly emphasize that while rising asset exposure remains the largest driver of the overall upward trend, there is increasingly clear evidence that climate change is strongly contributing to the severity and frequency of weather-related events - particularly non-peak perils such as floods, severe convective storms, and wildfires - which have driven recent insured loss totals well above historical averages.

Since the insurance industry is meant to help society by shielding it from the financial consequences of such events, offering insurance for affordable premiums is of great importance. Climate change and increasing exposure is therefore a growing concern for both the insurance industry and policyholders, as the affordability of insurance – and in some regions, even the insurability itself – of property is increasingly questioned. Insurers are on the front line of managing climate-related risks, yet the “tragedy of the horizon” implies that market participants may fail to internalize long-term risks. This creates not only financial exposure but also raises questions about the responsibility and potential liability of insurers and other financial institutions as climate risks become increasingly foreseeable, which is sometimes referred to as *responsibility risk*, a third category for insurers adding to transition risks and physical risks, see, e.g., [33, 152] and [19].

To contribute to the discussion on physical risks, this paper brings together recent ideas, arguments and proposed solutions that have emerged within the actuarial community on this topic. Our focus is on physical climate-related risks in the non-life insurance sector, particularly the coverage of NatCat risks such as floods, storms, landslides, droughts and wildfires. Climate-related physical risks occur as well in health-related perils, including heat stress and vector-borne diseases, and many of the arguments discussed later in this paper are relevant to these as well, but we refer to [138] and references therein for a more detailed account and description of climate-related changes in the insurance of such risks.

The paper is structured as follows. Section 2 summarizes the balance sheet approach to managing an insurance company from a regulatory perspective, discusses the general concept of insurability, and highlights the specific challenges faced by NatCat insurers in this context. Section 3 examines how climate change and increasing exposure affect and challenge these processes. Section 4 explores the role of regulation in addressing these challenges, while Sect. 5 looks into the role of prevention and mitigation of risks. In Sect. 6 we then discuss both solutions and "non-solutions" to go forward, in terms of the data collection, model development, adaptation strategies of insurers and reinsurers, as well as the contributions of states and regulators. We also emphasize the importance of adopting a multi-period and long-term perspective on climate-related risks. Finally, Sect. 7 concludes and provides some recommendations.

## NatCat insurance and its challenges

When discussing the pricing and insurability of NatCat risks, it is natural to consider the insurance activity from a solvency perspective. For that purpose, let us first recall more generally the (European) solvency approach for determining capital requirements of an insurance activity (for the clarity of exposition we take a number of simplifying assumptions in what follows).

### Solvency requirement of an insurance company

As for any insurance activity, an insurance company holding (or even specializing in) a portfolio of natural disaster risks needs to manage its liabilities and assets by following a balance sheet approach. According to the regulatory rules in most countries, one has to stochastically model all the liabilities and assets in the portfolio. In the language of European regulation (Solvency II), one then has to determine the present (market-consistent) value of assets $A_0$ as well as the present economic value of liabilities $L_0$. The difference (called risk-based capital) $\text {RBC}_0=A_0-L_0$ is the surplus (or free capital). One year later, the value of the assets will be $A_1$ and the value of the liabilities will be $L_1$, leading to $\text {RBC}_1=A_1-L_1$. The regulator then prescribes a minimal solvency requirement $\text {SCR}_0$ at time 0 for the changes within this year according to the model assumptions, i.e.,

1 $$\begin{aligned} \text {SCR}_0=\rho \left( \text {RBC}_0-\frac{1}{1+r_{0,1}}\,\text {RBC}_1\,\Big |\mathcal {F}_0\right) , \end{aligned}$$

where $r_{0,1}$ is the risk-less interest rate for one year, and ρ is the risk measure chosen by the regulator. Moreover, $\mathcal {F}_0$ is the filtration that signifies all the information available at time 0.

**Fig. 1:**
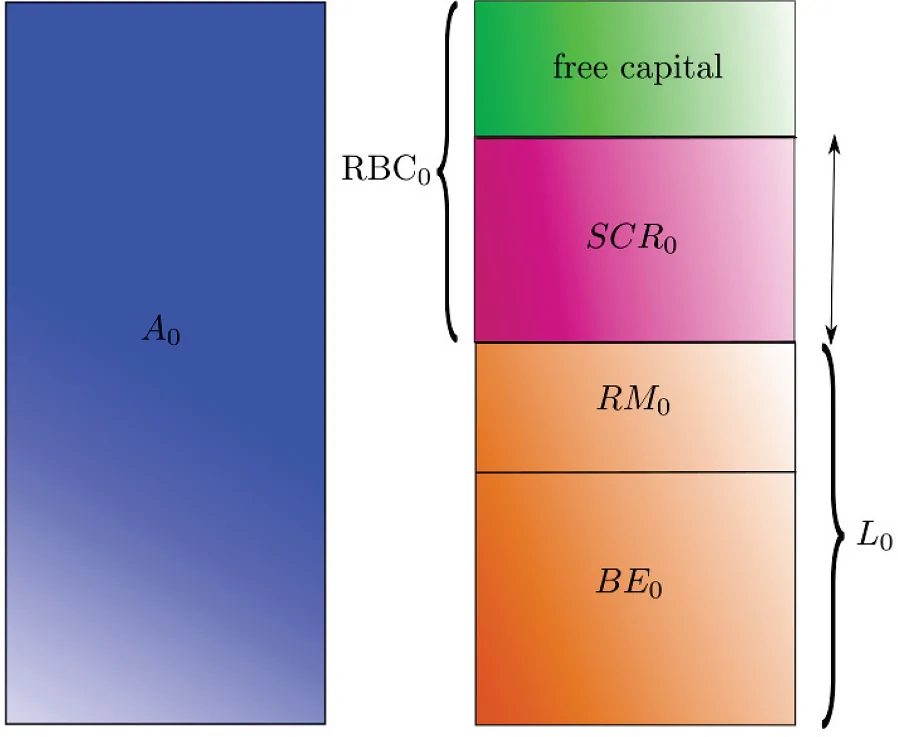
Balance sheet approach for a one-year time horizon

The company is considered solvent if $\text {RBC}_0>\text {SCR}_0$, and $\text {RBC}_0/\text {SCR}_0$ is called the *solvency ratio* (cf. Fig. 1).

The economic liability value $L_0$ is the amount that would have to be paid to another insurance entity to take over this risk. It sometimes contains a hedgeable part for which a market price may be available (see e.g. [54, 144]), but typically it has to be ’marked-to-model’. It consists of the expected claim costs (best estimate $\text {BE}_0$) plus a risk margin $\text {RM}_0$ which can be interpreted as the amount needed to attract shareholders for the risk bearing of the portfolio (rather than choosing other investment opportunities in the market). Denote by

2 $$\begin{aligned} S=\sum _{i=1}^{N}X_i \end{aligned}$$

the aggregate claim size in the portfolio to be paid during the next year (for simplicity paid at t=1), where $X_i$ are the random variables describing the individual claim sizes and *N* is the number of claims during this year. In order to focus on the insurance risk, let us now neglect any asset risk (and other risks such as credit risk, counterparty risk etc.), i.e. $A_1=A_0(1+r_{0,1})-S$, and also neglect potential new business at t=1. If we further assume that all insurance claims are settled within the year, then $L_1=0$ and one obtains from (1)

3 $$\begin{aligned} \text {SCR}_0=\frac{1}{1+r_{0,1}}\,\rho (S|\mathcal {F}_0)-L_0, \end{aligned}$$

from which it becomes clear that the solvency requirement is driven by the tail of the aggregate insurance risk (2). Indeed, the risk measure ρ is sensitive to the tail, as it is typically the Value-at-Risk $\rho (S|\mathcal {F}_0)=\text {VaR}_{\alpha }(S|\mathcal {F}_0) = \inf \{ x \in \mathbb {R}~|~ {\mathbb {P}}(S>x) \le 1 - \alpha \}$ (with $\alpha =99.5\%$ in Solvency II, cf. [65]) or the Expected Shortfall $\rho (S|\mathcal {F}_0)=\text {ES}_{\alpha }(S|\mathcal {F}_0) = \mathbb {E}(S|S>\text {VaR}_{\alpha }(S|\mathcal {F}_0))$ with $\alpha =99\%$ for the Swiss Solvency test, cf. [66].

For a given *S*, the relationship between the risk margin $\text {RM}_0$ and the solvency capital requirement $\text {SCR}_0$ is typically dictated by the expected return of the shareholders on their investment $\text {SCR}_0$:

4 $$\begin{aligned} (1+r_{0,1})\mathbb {E}([\text {SCR}_0+\text {BE}_0+\text {RM}_0-S)]^+|\mathcal {F}_0)=(1+r_{0,1}+r_{{CoC}})\,\text {SCR}_0, \end{aligned}$$

where $r_{{CoC}}$ is the cost-of-capital rate (the spread above the default-free rate $r_{0,1}$ for the more risky investment in the insurance business).^1^ Note that (4) contains the limited liability option $(\cdot )^+=\max \{\cdot ,0\}$, that is, the investor will not lose more than the initial investment $\text {SCR}_0$, even when the aggregate loss *S* exceeds $L_0+\text {SCR}_0$. If we drop that limited liability, then (4) simplifies to

5 $$\begin{aligned} \frac{r_{{CoC}}}{1+r_{0,1}}=\frac{{\text {RM}_0}}{\text {SCR}_0}, \end{aligned}$$

in which case one can directly interpret the risk margin $\text {RM}_0$ as the expected return in excess of the risk-free rate for the shareholders (which is also the rationale along which it is treated in Solvency II and SST).

If the insurance risks are not settled within the one year, then the risk margin at time 0 is in many regulatory frameworks determined by

6 $$\begin{aligned} \text {RM}_0=\sum _{t=0}^\infty \frac{r_{{CoC}}\cdot \text {SCR}_t}{\prod _{j=0}^t(1+r_{j,j+1})}, \end{aligned}$$

where $\text {SCR}_t$ are the projected solvency capital requirements at the future time periods *t*, and $r_{j,j+1}$ is the risk-free rate from time *j* to j+1. This formula suggests a constant cost-of-capital rate, which for risks with longer maturity might be a strong assumption, and a more prudent choice for capital allocation may be more appropriate in such situations, see for instance [5].

A main take-away from the above considerations is that - whatever insurance risk *S* the insurer is facing - it leads to an $\text {SCR}_0$ according to (3) and implied capital costs $\text {RM}_0$ according to (5) or (6). If one finds investors for the amount $\text {SCR}_0$ and the means for raising the amount $\text {RM}_0$ (typically through premium loadings, but see also [8] for how to potentially include risky investments in such considerations), then the insurance of the portfolio can be realized directly. If that is not the case, one needs to try to reduce the amount (3) by reshaping the risk *S*, cf. Sect. 6.

### Insurability

One may broadly define insurability from an actuarial perspective as the situation when the above determination of $\text {SCR}_0$ and $\text {RM}_0$ can be done, which then also determines the needed premium level (for additional classical criteria of insurability like randomness, measurability and absence of moral hazard, see e.g. [21]).

Correspondingly, there are situations under which actuarial insurability of the risks in the portfolio is *challenged*, namely if

- one does not have sufficient confidence in the distributions of $X_i$ and subsequently in the determination of ${\text {RM}_0}$ and ${\text {SCR}_0}$.^2^
- the risks $X_i$ are positively dependent, leading to large values of (3) (and the confidence in the understanding of the dependence structure may also be limited).
- the risks are non-identical (heterogeneous), in which case it may be complicated to find appropriate (’fair’) ways of subdividing the aggregate $\text {RM}_0$ into individual premium safety loadings (at this level, questions of solidarity within the society already may play a role).
- $\text {Var}(X_i)$ is not finite, in which case coefficient-of-variation considerations and intuition based on the central limit theorem are no longer applicable (but the risk is then still insurable according to the above criterion).

More dramatically, actuarial insurability is *endangered*, if

- the number *n* of insured risks in the portfolio is so small and/or
- the risks $X_i$ are so *strongly* dependent

that the resulting diversification benefits do not suffice to keep the solvency capital ${\text {SCR}_0}$ and the needed premiums reasonably low. In both cases above, insurability might theoretically be there from an actuarial perspective, but the necessary premiums are too high to lead to a practically implementable insurance scheme (see the section below for more details on the issue of affordability). Yet worse, it may happen that

- $\mathbb {E}(X_i)$ is not finite.

In that latter case, the risk is not insurable at all from an actuarial perspective (even the best estimate $\text {BE}_0$ is then already infinite, so that no finite premium value can be offered for the unlimited coverage of the risks).^3^ We refer to Sect. 6 for possible solutions in such situations.

One sees that for the judgment about (actuarial) insurability of risks, a profound understanding of the aggregate claim size $S=\sum _{i=1}^{N}X_i$ is key, both for one- and multi-period frameworks.

### Particular challenges for NatCat insurance

It is of interest to look into what makes NatCat risks special or particularly challenging in light of the above considerations. First, the determination of the distribution of the risks $X_i$ and their dependence is harder than for classical insurance lines, as there are *fewer historical data points* (otherwise the name ’catastrophe’ would not be justified). Secondly, the tails are often *very heavy*, which leads to additional challenges in the statistical studies of distributional properties (see e.g. [3] for a recent survey of extreme value techniques in the insurance context). Quite typically, the variance of the underlying risks is infinite, and sometimes even their expected value may not be finite (see e.g. [101, 135]). Additionally, when basing the analysis on historical data, it can be *complicated to make data points comparable*, as the exposure and the vulnerability change over time (e.g., additional houses in an area, the building material may have improved over time, previous catastrophes may already have damaged property, prevention and mitigation measures may reduce the claim sizes over time, etc.) and the environmental conditions can vary considerably from one location to another, so that a pure mixture modelling approach may be too coarse for the purpose. Furthermore, there is often *very strong spatial, and sometimes also temporal, dependence* of risks $X_i$. Additionally, due to different hazards and exposures, risks in a NatCat portfolio are often very *heterogeneous*, which is a challenge for offering fair premiums and coverage (see e.g. [25]), and the role of solidarity may enter more prominently than for many other lines of business.

The above conditions (in particular the very heavy tails and the strong dependence) make it typically harder to achieve sufficient diversification in order to reduce the solvency capital to a level for which affordable premiums can be offered and the insurability from an actuarial perspective also leads to a practically implementable insurance scheme (we again refer to Sect. 6 for possible solutions, including exposure limits, reinsurance, alternative risk transfer and considerations with respect to the time horizon). The diversification benefit strongly depends on the particular peril and on the region (not only where it is, but also how large it is!). While some quantitative guiding in that direction is offered to the insurance industry by vendor models, there are also a number of academic papers that assess the diversification benefit for a particular peril and region quantitatively, see for instance [7, 27, 44, 46, 112, 134]. In that respect, it is of interest to understand for which types of NatCat risks pooling is already efficient on the level of municipalities, provinces, individual countries or entire continents, and a lot of future research is still needed in that direction.

When the analysis of heavy distribution tails is based on a few observations only, it is well-known (see e.g. [20]) that the robustness of results can be quite challenging and even the proper attribution of large data points to the same or another event can have a considerable influence on the conclusions. For instance, several large data points tend to increase the estimated mean of an underlying distribution and lighten the tail, whereas fewer large values typically lead to the conclusion of very heavy tails. This becomes most apparent when one has to decide whether some large data points should actually be considered as one aggregate (even larger) point or not (two concrete examples in that direction are the attribution of the two years 2002 and 2003 of annual catastrophe fund claim data eventually assigned to one flood event [133] or the difficult attribution of claims from the two subsequent winter storms Lothar and Martin 1999 in France to separate events, cf. [108].

**Affordability of NatCat premiums.** Complementing the actuarial viewpoint, a crucial additional condition for insurability in practice is that the resulting premium amounts are affordable for the policyholders. Insurance is considered affordable when premiums remain within the financial capacity of households and firms while still reflecting the underlying risk. For NatCat risks with its often highly correlated and potentially extreme losses, this can be more challenging than for classical business lines, see e.g. [94]. Such pricing can result in substantial premium differences across regions and income groups, potentially making coverage difficult to obtain for high-risk or low-income households, see e.g. [126]. When premiums increase beyond households’ willingness or ability to pay, one faces underinsurance and widening protection gaps (see, e.g., [26, 58]). Possible solutions to maintain affordability while preserving incentives for risk reduction in such situations include public–private insurance schemes, cross-subsidization mechanisms, or government-backed reinsurance, see for instance [95, 115] and also Sect. 6.

## What changes with climate change?

As mentioned in the previous section, already under a priori time-stationary conditions of the underlying natural hazard it can be complicated to make data points of different events over time comparable (in terms of corrections for a different building stock (growth in economic assets and population in hazard-prone regions significantly amplifies loss magnitudes over time!), changing building material, changing building value index, inflation, potentially applied preventive measures to mitigate parts of the risk etc.). This challenge gets further exacerbated when the underlying hazards themselves change their risk characteristics over time in terms of frequency, intensity and spatial dependence structure. The overall goal to enable proper (re)insurance solutions for NatCat risks in terms of premiums and capital requirements (eventually, the ’updated’ quantities $\text {BE}_0, \text {RM}_0, \text {SCR}_0$) then still asks for the identification of the up-to-date distribution of the individual risks $X_i$ and its aggregate sum *S*. Once the distribution of *S* is available (or agreed upon), insurance can be offered along the same classical principles. But if an analysis over larger time horizons is needed (for instance in terms of projections on a country level for respective policy implications of governments), then one even needs to identify the distributions of the random variables $X_i(t)$ as they change over time. And, naturally, a certain robustness of the involved assumptions will be needed so that respective decisions are sufficiently backed up and therefore solid.

It is nowadays without doubt that the frequency and intensity of many natural disasters increase with time due to climate change, and in many cases significantly (see e.g. [150]). Clearly, each peril needs a profound separate investigation, and the severity of such changes also depends on the considered region.

A priori, there are two ways to consider this effect in the modelling. The first one is to pose assumptions on an increased frequency and increased severity of events (for instance through parametric assumptions on frequency and severity parameters in the respective distributions) and then adapt the previously used models accordingly. This may work in a satisfactory way when one has sufficient empirical insights (for instance in terms of historical evidence, or through a detailed understanding of increased air and ocean surface temperature on the underlying hazards) to challenge the respective assumptions. However, it may already be difficult to assess whether the current claim distribution of a particular risk is still of the same type as in the past (but with changed parameters) or whether for instance the tail of the distribution is even more profoundly reshaped. Respective conclusions can then typically not be taken anymore solely on the basis of claim experience (historical data points) and (possibly ad-hoc) assumptions. Also, the choice of the time window that is considered relevant for respective conclusions may be very difficult.

The second possibility can be seen as a change of paradigm: While for decades actuaries have been used to (at least in part) base their analysis on historical data points, for the study of NatCat insurance risks under climate change one can also decide to generate ’synthetic’ claim experience. Concretely, one can simulate *hazards*, model the *vulnerability* of property and use *exposure* information to produce resulting claim figures for the simulated hazard. This approach relies on a detailed understanding of relations between environmental random variables and the final resulting claim sizes, and often can only be achieved in an inter-disciplinary way collaborating with climate scientists and meteorologists. The simulation of realisations of the hazard can for instance follow from global climate models (GCM) for physical variables like temperature, wind speed, air pressure etc., down-scaled to regional climate models (RCM, like e.g. the Cordex for Europe, see [85]), and further down-scaled to a country- or even more granular level. A stochastic weather generator (like e.g. [74, 128]) can then generate the concrete environmental variables (like precipitation, winds, hailstones etc.), which can then subsequently be transformed into resulting financial losses for each simulation run, leading to synthetic claim figures, see also [121, 170] for other approaches. Note that certain aspects of the above procedure were and are also provided to the insurance industry by services offering vendor models (typically as a black box), but in the last few years the academic research community has made substantial progress in this direction and nowadays many transparent algorithms are shared. It should be noted, however, that proceeding in the above way is no trivial task and can lead to enormous methodological, computational and time-consuming challenges, and an inter-disciplinary team of researchers is typically needed. Systematic descriptions of catastrophe modelling as a multi-disciplinary discipline are provided by [77, 105, 107].

A particular advantage of this ’synthetic claim generation’ approach is that the spatial dependence between risks is automatically generated and does not need to be modelled with assumptions on copulas or random fields that may not be easy to substantiate. Also, changing climatic conditions can easily be considered by using different assumptions and scenarios on the underlying temperature and/or the GCM and RCM input (see e.g. [41] for a respective study on hail risks).

From an actuarial perspective, one can then aggregate the generated regional claims to larger units of a required size to assemble the claim size experience needed for the actuarial analysis. The resulting aggregate claim distribution *S* can finally result simply from the empirical (generated) datapoints, or one may again (preferably) invoke a statistical fitting approach on the obtained datapoints as is done in a classical analysis. And once the distribution of *S* is obtained, a procedure as described in Sect. 2.1 can be launched again to determine the premium and solvency capital levels that are necessary for offering the insurance coverage. The crucial question to be answered is then whether these levels are acceptable for all stakeholders in the process (policyholders, insurance company and regulator), so that the insurance solution can be realized. The regulator represents the state, and the latter has a strong interest in efficient insurance solutions, as otherwise the state has to take a more prominent role in finding alternative solutions, see Sect. 6.

In both approaches, one key challenge is to deal with a non-stationary context with high model uncertainty (see also [87]). Extreme weather event probabilities and severities are influenced by many major phenomena, often in parallel, see [69] and [100]. As an illustrative example, [76] state that for Brazil there are at least ten of these to consider, most of which correspond to oscillations of certain physical systems of the planet with very diverse periodicities (years for ENSO, but 50 days for Madden Julian Oscillations and decades for Atlantic Multidecadal Oscillations (AMO) and Pacific Interdecadal Oscillations), see also [40, 51]. Conjunction of extremal points of these cycles can then lead to extremely severe events, but such circumstances have only rarely or never been observed (see e.g. [34, 113]). See also [89] for how the occurrence of hurricanes is influenced by multiple teleconnections (concretely, ENSO and AMO). There is therefore a high uncertainty about future severities and frequencies of natural disasters in a world that will have very different characteristics from the conditions observed in the past.

Also, due to climate change, some cycles may become more prominent. As a case in point, in the above example, [76] note that ENSO is expected to be more prominent for the Brazilian climate problem of different cycles, and among the 21 models involved in the IPCC analysis only three or four reproduce correctly key features of the ENSO impact on Brazilian climate. While this leads them to recommend to use one of those three or four models, some other models may work better for other parts of the world.

It is therefore a delicate matter for international insurance companies and reinsurers to select a relevant climate model to aggregate risk or to use combinations of models or direct outputs of IPCC if the appropriateness of three fourths of the panel of models are already questioned in some parts of the world by atmospheric studies specialists. It is even harder to precisely assess the impact of potential tipping points (like for example a major oceanic current vanishing) or of "temporary" circumstances on climate in different regions (like a cold wave following a large volcano eruption, for example). For an interesting recent study on the anticipation of tipping points in the context of transition risk, in particular the appearance of doubtful incentives of increasing emissions before its arrival and therefore accelerating their arrival, see [49]. Even if significant uncertainty remains regarding both the timing and threshold of a tipping point of the Atlantic Meridional Overturning Circulation (AMOC), reflecting limitations in climate models and sensitivity to assumptions on forcing and system dynamics, [57, 165] and [163] anticipate that a collapse or strong weakening of the AMOC would have profound global impacts. In particular the impacts on Europe would include rapid regional cooling, shifts in precipitation patterns, and increased frequency of extreme events (notably cold extremes), with potentially severe consequences for water resources, agriculture, and socio-economic systems.

In parallel with climate change impacts, new external massive datasets have become available, together with more computing power to run Artificial Intelligence (AI) tools. Data and AI provide the insurance and reinsurance industry with more means of potential risk discrimination, which could reinforce the risk of demutualisation and exclusion for risks triggered or worsened by climate change, like clay shrink-and-swell (RGA) risk in France or coastal flooding risk due to sea-level rise, cf. [9, 80] or also [16, 35, 53]. This sheds another light on the insurability issue, as protection gaps may grow in many regions of the globe where certain houses could become uninsurable without intervention from regulators and states.

Besides the actuarial aspects of insurability, the affordability of (typically increasing) premiums for NatCat policies under climate change and increased exposures is another crucial challenge, cf. [94, 106]. With risk increasing, risk-based pricing-while economically efficient and necessary for insurer’s solvency-can lead to situations where affordability is lost (see e.g. [98]). This can result in declining coverage rates in areas most exposed to natural hazards [156], and correspondingly increasing widening climate insurance protection gaps [61]. See Sect. 6 for potential ways to deal with this issue.

Note that the considerations in this section focussed on a one-year time horizon, which is the time horizon underlying the typical balance sheet approach and the main concern of the regulator, so that efforts are concentrated on the identification of the underlying risks within this time frame. Changing conditions over a longer horizon suggest, however, a longer-time view in terms of business model, prospects and longer-term planning, see Sect. 6.4 for more details.

## The Role of Regulation

In general, the first role of the state is to provide assistance before, during and after natural disasters, to seek international aid if necessary (and if possible, as international tensions may complicate international cooperation, and several countries or continents may be in difficulty simultaneously due to climate change), and to be the insurer of last resort (again, if possible). See, e.g., [94, 148, 159, 177] for some general accounts on this topic.

Regulators, as well as many actuarial associations, have first approached climate-change following the methodology of the Task Force on Climate-related Financial Disclosures (TCFD), which has played an early important role in structuring the identification, assessment, and disclosure of climate-related risks and opportunities (see [68]). Adopting a similar framing of climate-related risks, the International Association of Insurance Supervisors extended the discussion to supervisory and insurance-specific considerations (cf. [84]). By providing a framework for governance, strategy, risk management, and metrics, this has supported the integration of climate considerations into financial decision-making, actuarial modelling and pilot exercises of several regulators.

**Government as insurer of last resort.** The mere fact that people believe that the state will act as an insurer of last resort may harm prevention and insurance efforts [28, 137]. In order to avoid this, the government may impose obligatory solutions. This is the case for example in France [37], Turkey [78], Romania [118] and (in large parts of) Switzerland [149]. Obligatory solutions represent a way to impose mutualisation and solidarity [127]. When times become more difficult, more affluent groups may be tempted to opt out of the group [145]. This occurred at country level in Belgium and Spain where Flanders and Catalunya, respectively, wanted to get out (even if the wealth of these provinces was not the only reason). In the absence of an obligation, policyholders living in areas less impacted by natural disasters and climate change are tempted to stop sharing risks with policyholders exposed to such perils.

**Solidarity and fairness.** Obligatory insurance is one measure to enforce solidarity. It is then a natural challenge how to share respective risks between individuals, companies or regions, e.g. in terms of cross-subsidizations in premiums. For a modern account of fairness in the actuarial context see [38]. Recent regulations like the AI Act in the European Union may help individuals to be better protected from extra-discrimination [164]. But the real question is how regulators will shape the future of "classical actuarial discrimination", which works well if some individuals are 5 times riskier than others, but needs some complement or mitigation if some individuals are 10,000 times riskier than others.

This connects also to the literature on economic capital allocation. If pricing incorporates risk margins, seen as an allocated cost of economic capital [55], for instance computed with an Euler allocation principle, then the very risky policyholders would see their premium increase a lot and could face non-insurability [96].

Should one set a maximum premium level, or impose insurance companies to smooth patterns of their pricing algorithms in order to achieve an acceptable level of "fairness", which still takes (part of) the risk discrepancies into account but guarantees inclusion? Classical references for such questions in the field of economics are, e.g., [43, 88, 139].

**Proactive Legal Adaptation.** Laws must evolve to address the non-stationary risk environments and climate-induced volatility without any unnecessary delay, rather than relying on historical norms. One further option consists of building up a reserves fund. This was successfully implemented by the state of Marocco, which protected uninsured people thanks to a fund dedicated to three perils, including earthquake risk [161]. The fund became operational and a reinsurance coverage was signed just before the 2023 Al Haouz earthquake. In the context of climate change, there may not be enough time for that, and this mechanism is not viable in case of repeated events whose frequency increases too much.

Climate change and natural disasters affect not only individuals but also companies. Italy recently passed a law to make NatCat insurance obligatory for enterprises, effective for large companies since April 2025 and for smaller companies by the end of 2025 [140].

Should regulators include municipalities and regional authorities in their scope? For several recent events like the 2024 Valencia region floods [131] and the January 2025 Southern California wildfires, risk response failures have increased the number of human casualties and the total insurance loss. Enforcing relevant response systems and imposing fit-and-proper constraints to decision makers similarly to what exists for board members of insurance companies could help improving prevention and mitigation strategies, early-warning systems, crisis management and disaster recovery.

There can also be unintended consequences of price regulation. As a case in point, in California regulatory constraints on insurance pricing-aimed at protecting consumers-have limited the ability of insurers to fully reflect underlying wildfire risks in premiums. Over time, such constraints weakened risk-based price signals, thereby encouraging development in hazard-prone areas and increasing exposure. This dynamic has contributed to growing insurability challenges, with several major insurers reducing or withdrawing coverage in high-risk regions as losses have escalated [31, 86]. More broadly, regulations that distort risk-based pricing may undermine one of the core functions of insurance markets-namely, the efficient pricing and signaling of risk-and can inadvertently lead to greater risk-taking and accumulation of losses, cf. [92].

States and regulators should promote insurance and risk education to the general public and to decision-makers in order to raise awareness, preparedness, and to reduce the protection gap, see e.g. [24, 114, 117, 119, 146].

States and regulators could protect the environment and reduce claim frequency and severity for some perils like wildfire. In some countries, after sections of protected land burn, it is said that there is nothing more to protect, and the land becomes available for agriculture or construction. Even if wildfires are becoming more frequent and more intense due to climate change, according to [172] up to 75% of the wildfires on the planet are human-made, partly intentionally to appropriate land. A law that guarantees that protected land will remain a protected area even if it is burnt by fire could in such a case be helpful.

It would be desirable to anticipate regulation adaptation. Otherwise, states and regulators may have to overreact to future exceptional natural disasters (made possible by climate change), shocking the society with sudden regulation changes (cf. [91, 142]). This anticipation would also reduce the correlation between physical risk and transition risk [23, 111].

Another approach is to impose a kind of social security for natural disaster losses or to have only one monopoly insurer or reinsurer. This may take several forms (a state reinsurer like CCR in France with obligatory reinsurance for all insurers through the design of a NatCat system, or a monopoly insurer like in many cantons in Switzerland). While efficiency and innovation may be questions in the case of a monopoly, a guaranteed clientele over a long time horizon can lead to more incentives for prevention.

Finally, regulations that reduce exposure (such as floodplain zoning and land-use planning) and those that reduce vulnerability, including building standards and retrofitting requirements, play an equally important role in shaping disaster losses and insurance outcomes, see, e.g., [29, 154]. Empirical studies suggest that the effectiveness of insurance-based incentives for risk reduction depends on the regulatory environment, particularly the presence of building codes and land-use restrictions that limit development in high-risk areas [82]. Consequently, disaster risk governance increasingly emphasizes integrated approaches combining insurance markets with spatial planning, infrastructure investment, and building regulation in order to reduce losses and maintain long-term insurance affordability, cf. [42].

## The role of prevention and mitigation

In the presence of worsening risks, insurability is threatened: in 2015, the former CEO of AXA made the influential statement that a ”4 degree world would not be insurable” and that changes in the behavior of both companies and population were necessary [50]. Indeed, prevention and mitigation appear to be a very useful tool to reduce climate-related risks and promote inclusive insurance in the future. In actuarial terms, one could interpret prevention and mitigation as reshaping the distribution of the aggregate loss *S* towards a more favorable – and better insurable – one. The costs for this reshaping then plays the same role as a premium for a reinsurance contract (which is another means of reshaping *S*). There are different types of prevention and mitigation (before or after the event, affecting either frequency or severity of events, or both). The substitutability between insurance and prevention has been discussed in the seminal paper of [59]. [71, 72] study the optimal prevention effort that an insurance company should make per unit of time in a risk theory context. In the case where prevention reduces the claim frequency for all claims, under mild assumptions, the optimal prevention effort level is quite robust: it does not depend on the surplus level (corresponding to the solvency ratio in Solvency II) nor on the objective (maximizing dividends up to ruin or minimizing ruin probability). If prevention however impacts only the frequency of some types of claims, then the situation depends on the surplus level. If prevention works only for large risks, it may happen that insurers with lower solvency ratio reduce their ruin probability by implementing less prevention, as it is important for them to get out of the "danger zone (low surplus)" as quickly as possible. For some lines of business (concretely in France for instance), prevention efforts are considered by some insurance companies as part of their corporate sustainability responsibility and not really part of their claim frequency management strategy, as competitors may benefit from their prevention efforts after their clients lapse, see, e.g., [99]. For classical insurance economics references on the role of prevention in a NatCat context, see [56, 93, 96]. For a review on how insurance programs are linked to risk reduction and prevention incentives for NatCat risks, see [116].

As mentioned in Sect. 4, a monopoly may encourage more prevention and mitigation efforts [45]. If one wants to maintain efficiency and competition between insurers, another solution could consist of imposing a minimum proportion of insurance premiums dedicated to prevention. In France, the state reinsurer CCR also invests into prevention on the asset side of the balance sheet [30]. In Spain, there is also a mandatory NatCat coverage system, where insurers collect add-on premiums and for ’extraordinary’ risks a public insurer pays the losses directly, builds up reserves and seeks reinsurance coverage. In terms of prevention, this public insurer is not involved in a financial way directly, but participates with technical expertise, loss data and prevention governance, see, e.g., [18].

For natural disasters, very often prevention by the policyholder does not prevent the event to occur, but may limit the consequences (*mitigate the risks*). Mathematically speaking, in terms of costs and benefits the optimal prevention problem then becomes closer to an optimal reinsurance problem (see e.g. [4]).

One of the most undisputed examples of mitigation in terms of claim severity reduction and in terms of keeping more contracts insurable corresponds to early warning systems for floods following heavy rain. In France, Predict (a spinoff of the national weather agency Meteo France) offers early warning services that enable small businesses like garages to have two hours to relocate their most precious goods (like expensive cars in the case of garage) to safe places before the water arrives [132]. For partner insurers, even if the event probability is multiplied by 3 or 5 due to climate change, risk may remain insurable because the severity of the claim is divided by 20 or 50. Early warning messages to policyholders in case of arriving natural hazards are also becoming more common in other countries (e.g., thunderstorm and strong winds with hail warnings from cantonal building insurers in Switzerland, cf. [149]). For general accounts on the role of early warning systems in disaster risk reduction, see, e.g., [123, 143].

Prevention may be implemented before or after the claim: on the French Atlantic coastline, the state already proposes to purchase some houses that will soon be threatened by sea-level rise (cf. [36, 81]). In Denmark, houses that have been flooded have to be rebuilt further away from the coast [17]. This type of prevention and adaptation efforts is of course complex, involves many social and economic issues, and requires planning at the state level, with the participation of the insurance and banking industries, see [158] for some general considerations.

The role of psychology in the perception of climate change and its risks is also an important element, see e.g. [73, 83]: climate change anxiety is rising, and risk attitudes are decoupled in a long-term, uncertain context. Risk and insurance education efforts must be coupled with attention to psychological impact, also preparedness is key. Some events are likely to take place in new regions for the first time due to climate change. This is for instance the case for hail, due to the change in atmospheric conditions. In Baltic countries, for example, inhabitants who were not familiar with hail recently went outside to protect some goods and suffered injuries from large hailstones [97]. Improving preparedness is more difficult for risks that never occurred than for risks that the population is familiar with but whose consequences worsen or which become more frequent [93, 124, 151].

Another aspect of psychology concerns the risk of substitution between prevention and insurance investigated by [59]. The risk of incentivizing idleness (in terms of adaptation and mitigation of climate change) due to state protection (subsidizing affordable insurance or acting as a payer of last resort) has been a concern for regulators and public decision makers for several years (see e.g. also [157] for a discussion of charity hazard). Independently of the feasibility for the state to play this assistance role in the long run, in the presence of climate change some stakeholders believe that individuals must face part of the consequences in order to have a real incentive to adapt behaviour.

To illustrate how this debate relates to actuarial concerns, one may consider agricultural insurance (cf. [15] for a recent survey). While climate scientists typically rely on comparisons with a 30-year average to determine whether a given year corresponds to an extreme event in terms of precipitation or drought, many insurance compensation schemes instead use a so-called "olympic mean" computed over a shorter time horizon. For example, the highest and lowest observations over the past five years may be excluded, with the average then taken over the remaining three years; compensation is subsequently determined by the excess of a new observation over this mean. Although trimming increases stability in both approaches, the shorter time horizon implies that large observations under recently changed conditions appear less extreme, thereby reducing compensation. At the same time, this approach keeps risk levels more manageable for insurers and - according to some decision-makers - encourages policyholders to adopt prevention measures and climate adaptation strategies more proactively. Naturally, such an argument is somewhat controversial.

Of course, most insurers and reinsurers have also started to quantify their carbon footprint and try to reduce it. This corresponds to another type of prevention of climate-related risks in the long run. The reasonable use of AI, which requires electrical power, water and other natural resources, should become a hot topic in the near future [52, 153, 173].

## Solutions and non-solutions

In this section, we discuss solutions and "non-solutions" for different items: data and models, insurers’ and reinsurers’ adaptation, as well as state and regulatory bodies strategies. We also discuss the choice of the time horizon.

### Data and models

One purpose, one model: this key statement is particularly relevant for the analysis of climate-related risks. Indeed, models and indices have been provided for years by well-established international groups of specialists, and it would be impossible or extremely costly for an insurer (or even a reinsurer) to try to compete with these groups. However, the available models or indices have been developed for a special purpose, and one must not forget that many issues may arise when re-using those models as a building block of another model for a different aspect, or at a more local scale, or from a different perspective.

To illustrate the risk of misuse or misinterpretation, let us start with IPCC RCP’s and SSP’s. They are generated thanks to the average of 30+ models, based on the idea that some sources of uncertainty are "neutralized". A first example of misinterpretation of IPCC methodology is that most insurers and reinsurers consider RCP8.5 (or SSP 5−8.5) as one of the most extreme scenarios, although several uncertain risks (including the jet stream slowing down or vanishing) are not present in this scenario. It is therefore needed to complement this first set of stress tests produced from IPCC RCP’s and SSP’s with additional scenarios in which some tipping points are reached (see e.g. [13]).

Also, as mentioned earlier, among the 30+ models used in IPCC, only four represent correctly some key characteristics of the Brazilian climate related to ENSO and to Madden-Julian Oscillations. Averaging these four models with the 29 other ones would no longer make sense for their studies about precipitation extremes in Brazil. In other parts of the world, probably some other models used in the IPCC model panel are more relevant, which makes it hard to select one model for the entire world and to aggregate risk at global level. We would also like to mention that RCP 8.5 is widely no longer considered plausible from an emissions perspective, and the IPCC is moving towards the introduction of Representative Emissions Pathways (REPs) with more extreme scenarios around fundamental uncertainties that arise from the carbon cycle and climate feedbacks, cf. [103].

Another example concerns climate risk assessment for some particular regions. It could be tempting for an actuary to use a local version of the Actuarial Climate Index (ACI), an index developed initially by North American actuarial associations for USA and Canada, and later adapted to some European countries, cf. [70, 176]. According to the ACI website, *the Actuaries Climate Index is an objective measure of changes in extreme weather and changes in sea level relative to the base period of 1961 through 1990. The Index is an educational tool designed to help inform actuaries, public policymakers, and the general public on changes in these measures over recent decades.*

These ACIs can be convenient and provide relevant aggregated information about past climate change in a country or region with weights adapted to the insurance industry perils, and sometimes the correlation of these indices to actual perils can be satisfactory, see e.g. [175, 175]. However, one must be very cautious to use ACIs as a general off-the-shelf tool for assessing climate-related hazards, as the averaging over several perils may blur signals and identified correlations may easily change over time. Accordingly, using such ACIs to create parametric insurance schemes to price a particular climate risk or to create some prospective scenarios may not be advisable. One may rather want to rely on more customized indicators for the concrete hazard under study, ideally in collaboration with specialists for the respective natural phenomenon.

Many large insurers and reinsurers are currently working with an interdisciplinary team on different aspects of climate change impact modeling. Some studies consist of developing an in-house model targeting a special peril in a special region, for which available models do not do a sufficiently good job. This is not new, and it was already the case 30 years ago when the insurance industry was challenging NatCat vendor models [75]. What is new is that the alternative approaches now absolutely need to take into account global circulation model features [32, 60]. The complexity and the chaotic properties of these models (removing a small mountain can change the climate of an entire continent) are tough challenges, on top of computational time issues [22, 120].

Interesting research projects already implemented in the insurance industry include works that aim at replacing complex climate models by some deep learning counterpart in order to save computation time, as well as projects that aim at generating more extreme events thanks to generative AI techniques (see [12, 104]) or thanks to synthetic circumstances obtained by shifting some cycles in order to create unseen conjunctions. Some past events (typically hurricanes or floods) can then be "replayed" and financial consequences for the insurer can be assessed in what-if scenarios like "what if the hurricane was formed in the atmospheric conditions of the day before or of two days after?", or "what if this other cycle was at its peak when the flood occurs?". The same methodology could be applied to assess potential impact of climate change or to get better information about the concept of "maximum probable loss", often used in reinsurance or risk management.

While satellite images have already been used for several years in order to assess risk or define a parametric insurance or reinsurance index (for agriculture insurance for example, cf. [168]), new satellite data (including LIDAR and INSAR) could be exploited in the future to improve early warning systems, index construction and risk assessment, and ultimately prevention, at the cost of dependence of some insurance industry value chains on the future availability of these satellite data.

### Insurers’ and reinsurers’ adaptation

With the development of satellite data, parametric insurance could be developed in order to provide fast payments in zones where sending an expert is too costly. NatCat triggers and indices should of course be carefully designed in order to keep basis risk (the difference between the outcome of the parametric trigger and the actual loss experience) as low as possible. Cat bonds could be developed in collaboration with the World Bank in order to protect fragile populations in the absence of concentration of wealth according to traditional insurance standards. One aspect to consider in that respect is, however, that with climate change there is potentially higher correlation between the Cat bond risks and classical market risks, so that the appetite for investors in Cat bonds may not be as high as in the past (when they could rely on a respective independence assumption; investors may for instance have long-term positions in stocks or bonds of companies that are threatened by physical risks). A careful design of the bonds would have to consider these stronger ties between assets and liabilities, in order to still satisfy the needs of all parties.

From the point of view of the insurers, affordability of premiums can be achieved in various ways, for instance through adding additional (sufficiently independent) insurance policies in the portfolio which leads to diversification benefits (and consequently reduces (3)), or through the introduction of policy limits, deductibles, reinsurance and alternative risk transfer solutions (like insurance-linked securities, see, e.g., [14]). One needs to keep in mind, however, that deductibles and policy limits leave the risk somewhere else and it depends on the concrete peril and situation whether this solves the issue for society to a sufficient extent. Also, a shortening of the reference period in parametric insurance contracts (like the 5-years olympic mean instead of the 30-year average of meteorologists discussed earlier) reduces premiums and generates stronger incentives to adaptation and prevention, but reduces the insurance coverage as well.

If the premium increases but remains in a range that is acceptable for the policyholders, then a slight mitigating effect may exist in case of increased frequency: less destruction may occur the second time a peril hits the same region as nothing is to be destroyed anymore before reconstruction (there might, on the contrary, also be some spillover effects for some perils). Besides, either the whole distribution becomes more extreme, or formerly extreme claims become more frequent, large claims with some kind of levelling. In the latter case, with more data and less "extremes", it might be easier to insure even if the premium increases, as the variance to mean ratio decreases. If claims occur regularly, this also encourages people to stay insured, and to keep prevention habits. But it is of course possible that the claim distribution worsens for some risks to such an extent that the "infinite mean" domain is approached or even reached, see e.g. [48, 90] and [109], and therefore posing insurability challenges, see the discussion in Sect. 2.

Reinsurers could raise reinsurance premiums or raise deductibles (which both happened recently quite significantly in the US and in Europe, see e.g. [10]), but of course then more risk stays with first-line insurers. In the past, reinsurance underwriting cycles emerged from the interaction of capital flows, loss experience, delayed information and behavioral feedback, as well as macroeconomic factors (see e.g. [102, 160, 169]). Even if changes from soft to hard market phases could sometimes be triggered by a large event, there was little particular correlation between the *current * cycle phase and the *current * claim intensity and severity. In a climate change setting, where ENSO oscillations may become the dominant NatCat modulating factor for some perils in certain regions, cycles may be perturbed, and one may face hard market situations during years where risks are at their peak, which could cause extra difficulty to find affordable reinsurance in hard phases.

Insurability can always be guaranteed from the insurer’s perspective through coverage limits, but then the society is not fully protected, which passes the problem on to the state and regulators (see Sect. 6.3 below).

If all risks are correctly identified and observable, and if premiums can be charged that are fair relative to the underlying risk, then the additional variability of the insurance portfolio (arrival of catastrophes, but then again calmer periods etc.) can in fact also be attractive for shareholders, see for instance [1, 2]), where optimal dividend strategies for NatCat insurance companies reveal a competitive advantage to classical insurance business lines (even when anticipating climate tipping points). The challenge in practice will, however, be a considerable degree of remaining model uncertainty.

For the society at large, if insurance solutions can be offered for an adequate premium, but these premiums are too high for certain individuals, then one may look into the efficiency of subsidizing such premiums. This may especially also be one way to deal with regions that are commonly considered uninsurable because the premiums are too high. A core question in that respect is then whether this is more efficient than tax-money financed catastrophe fund solutions which are currently implemented in some European countries, and how to otherwise deal with risks that may be considered uninsurable, see Sect. 6.3.

### States and regulators

Most states and regulators pursue the objective of (by all means) providing insurability to the population, for prosperity and welfare of the society. One possibility to try to increase insurability is to introduce a NatCat tax, with all challenges and difficulties related to such a new tax.

Other options include international solidarity and pooling mechanisms. The European Central Bank (ECB) and the EU regulator EIOPA have published a consultation paper on the opportunity and potential design of a European NatCat system, cf. [62]. The goal is to address the existing protection gap (which in Europe is estimated to currently be in the range of 75%) that could further grow in the coming years due to climate change and due to the availability of new datasets (see also [147]). See also [122] for a survey of the currently implemented systems in Europe.

One of the most promising ways to maintain inclusion and insurability are Public-Private-Partnerships (PPP), which can take very diverse forms. PPPs can involve households, insurers, reinsurers, and governments. Private insurers can provide and administer primary coverage, while reinsurers and international capital markets absorb part of the higher loss layers, and governments act as insurers of last resort or provide post-disaster compensation mechanisms, cf. [125]. These institutional arrangements vary widely across countries, from compulsory insurance pools to state-backed reinsurance schemes. Governments and municipalities play a crucial role through land-use planning, building regulations, and infrastructure investments that influence underlying risk levels, while regulators and supervisors shape solvency requirements and market design [62, 136]. At the same time, insurers can incentivize risk reduction through pricing and underwriting conditions, although strong risk-based premiums may create affordability challenges for households [82]. These interactions highlight the importance of aligning incentives across stakeholders, as decisions made in urban planning, infrastructure investment, and climate adaptation often determine the risk exposure that insurers and property owners ultimately face [42]. This is particularly relevant for prevention initiatives, because very often the state or national institutions (social security, national aid systems, national funds etc.) also benefit from prevention efforts. It makes sense for the states to partner with private insurers and to reward them if they can prove that their prevention effort paid off for the state as well (which may be tricky, as attributability in general is difficult, and it is even more difficult for climate risks).

As discussed earlier, as prevention and mitigation play an important role, regulators and states could impose that a part of the charged premiums needs to be dedicated to prevention in an obligatory fashion. A similar measure could also be taken for the asset side of the balance sheet (to encourage or impose investment of a minimum part of the assets into prevention initiatives).

Finally, when assessing future insurability of NatCat risks, it will be crucial to distinguish between the different perils, as they will not be subject to the same degree of changes. For Europe, the IPCC report [150] indicates that heavy precipitation events are projected to intensify and become more frequent across most European regions, increasing the likelihood of pluvial and river flooding. At the same time, coastal flood risk is expected to increase due to the combined effects of sea-level rise, storm surge, and heavy rainfall, particularly in low-lying coastal zones. Wildfire risk is projected, however, to increase most intensely in southern Europe, where higher temperatures and more frequent drought conditions are expected to intensify fire weather and extend the fire season. For wind-related hazards, projections are more uncertain: while extratropical storms remain a major driver of insured losses in Europe, climate models show mixed signals regarding future storm frequency and intensity. These differences in projected hazard trends highlight that the future insurability of climate-related risks may vary substantially across perils and regions, underscoring the importance of considering hazard-specific developments when discussing long-term insurance affordability and risk management.

### Single-period vs. multi-period view

We have already discussed the importance of how far to go into the past in order to calibrate models (in reference to the different cycles that affect climate, including inter-decadal ones) and to define the reference year for claim payment triggers (like the use of olympic means vs. a 30-year reference as in meteorology). Another key aspect of climate change modelling and the resulting management issues is the future time horizon.

Going back to the solvency perspective (cf. Sect. 2), Solvency II and other insurance and banking regulations are known to be "myopic", i.e. they focus on the short-term horizon, typically one year. Even if initial solvency regulations (in Scandinavia and then in Europe) were based on long term ruin theory computations, and even if multi-period liability valuation is a topic of its own in the actuarial research literature (see [5, 63, 64]), most insurance and reinsurance companies no longer calculate a ’long-term economic capital’ and only compute a short-term economic capital related to the 1-year horizon of Pillar 1 of Solvency II. The pronounced seasonality of many NatCat risks makes time horizons shorter than a year clearly inappropriate; at the same time, the rapidly changing profile for such risks over the years may give a comforting view on restricting to one-year considerations. However, a longer-term view may also be natural for various reasons, a sound business plan only being one of them.

Pillar II of Solvency II includes the ORSA (Own Risk and Solvency Assessment), which contains a set of scenarios usually limited to the duration of the business plan (three to five years). Recently, supervisors like ACPR in France encouraged insurers and reinsurers to assess climate change risks thanks to scenarios until 2050. Many insurers and reinsurers start to develop scenarios for some precise perils up to 2075 or even 2100. Main challenges include the level of uncertainty and the difficulty for decision makers to take into account these scenarios in their decision making process, as well as the design of such scenarios (see [167]). From an Enterprise Risk Management point of view, it is also a danger to focus only on climate change risks at this time horizon, as there are many other risks that should be considered as well.

The WEF 2024 risk map report [171] also illustrates the myopic view of many stakeholders: in spite of clear awareness of all stakeholders (at the 10-year horizon, environment, biodiversity and natural disasters clearly dominate while top risks are very diverse at the 2-year horizon), most risk management actions target non-green objectives due to short-term prioritization.

That being mentioned, the question is which actions could be undertaken to counterbalance this short-term view. In addition to accounting changes, prudential regulation changes could include lower quantiles and a longer time horizon for solvency requirements. One could for example use a $\text {VaR}_{95\%}$ instead of $\text {VaR}_{99.5\%}$ (or work with expected shortfalls in a similar way) at a longer time horizon (3 or 5 years). This would also encourage a more quantitative modelling of time diversification for NatCat risks, which is one further way of reducing the amount of solvency capital, and consequently premiums. Depending on how such a step is designed concretely, this would not necessarily have to be at the expense of a reduced safety level, and modelling at a 95% quantile level may be more reliable than for the classical 99.5% level. The analogue of lowering the quantile in expected shortfall calculations (used in the Swiss Solvency Test) would not benefit from that latter advantage, but would also help towards a more prudent view of NatCat risks and raise incentives for a long-term view. Enlarging the pooling potential over time (rather than space) has always been in the toolkit for risk management of NatCat insurers, and, depending on the considered time horizon, may also be beneficial for shareholders (see e.g. [47]). At the same time, firmly adopting a longer time horizon would introduce its own challenges. It would entail higher capital requirements to cover expected losses over the period of contracts, while reducing the flexibility for insurers to adjust to evolving loss profiles and changing physical and socio-economic conditions – factors that, as discussed earlier, have contributed significantly to rising losses over the last years. This, in turn, may create incentives for insurers to favor short-term risks, potentially leading to higher premiums for longer-term, climate-related exposures. There is therefore no straightforward optimal balance in addressing these trade-offs, and viable solutions are likely to remain context-specific, varying across regions and perils. For a general account of pooling principles over space and time, see also [67]. Recently, the regulation trend in the European Union is, in any case, more focused on the revision of the SCR levels for NatCat perils: increasing SCR levels for risks worsened by climate change, and considering the inclusion or the monitoring of new perils (wildfire, drought, etc.).

## Conclusion and outlook

In this paper we outlined key challenges related to the insurance of climate-related risks and explored potential societal solutions to maintain effective insurance coverage. Such solutions require efforts from all relevant stakeholders. We highlighted the role that prevention, mitigation and regulation can and should play in this context. The focus of the paper was on physical risks, particularly NatCat perils within the non-life insurance sector, although many of the arguments are more broadly applicable.

Climate change raises distinct but interrelated challenges for insurers, regulators, and governments, particularly in the presence of increasing non-stationarity of risks, growing protection gaps, and potential limits to insurability. Insurers must invest into targeted prevention and adapt their modelling frameworks and underwriting practices, while regulators face the need to ensure financial stability and consistent disclosure standards. Governments, in turn, play a key role in maintaining the insurability of risks through adaptation policies, public-private partnerships, creation of prevention reward mechanisms, protection of key water and energy infrastructure from vulnerabilities, as well as international collaboration in a tense geopolitical context.

Actuarial research has a critical role to play in developing forward-looking models, improving climate scenario analysis, and better quantifying uncertainty and insurability constraints. It is essential to link considerations to the latest available climate projections, see for instance the very recent CMIP7 analysis [162]. There is also a need for stronger interdisciplinary collaboration to bridge gaps between climate science and actuarial applications. It seems to become increasingly important to treat Earth as a system.

In recent years, international actuarial associations have already played a central role in advancing methodologies for addressing climate-related risks, particularly through the development of scenario analysis frameworks and guidance on integrating long-term uncertainty into actuarial practice. In the years to come, the training of future actuaries must evolve to incorporate climate-related risks more systematically, with greater emphasis on scenario analysis, decision-making under uncertainty, and treatment of data from multiple sources, including satellite data. Together, these efforts are essential to ensure that the actuarial profession remains equipped to support risk management and financial resilience in a changing climate.

## Footnotes

1 A typical choice for $r_{{CoC}}$ is 6%, although in the European Union it will be reduced to 4.75% at the latest in 2027. Originally, it was chosen in terms of typical values of the market β and the equity risk premium of insurance companies, see also [6] for a discussion on a justification of particular values.

2 In practice, it is common to augment a basic model for the risks $X_i$ by adding a (mixing) component of worst-case scenarios, and the respective assumptions underlying a respective scenario generator may be considered too arbitrary.

3 One may argue that, due to the finiteness of property values, $\mathbb {E}(X_i)$ can never be infinite, but the actual upper bound can be so large that this distinction does not make much practical difference (and is not detected by statistical means), as the stochastic behavior of the portfolio in any case then will start to mimick the problematic fluctuations of infinite-mean risks, see e.g. [39].
